# Rethinking competence in marine life cycles: ontogenetic changes in the settlement response of sand dollar larvae exposed to turbulence

**DOI:** 10.1098/rsos.150114

**Published:** 2015-06-24

**Authors:** Jason Hodin, Matthew C. Ferner, Gabriel Ng, Christopher J. Lowe, Brian Gaylord

**Affiliations:** 1 Hopkins Marine Station of Stanford University, Pacific Grove, CA 93950, USA; 2 San Francisco Bay National Estuarine Research Reserve and Department of Biology, San Francisco State University, Tiburon, CA 94920, USA; 3 Bodega Marine Laboratory and Department of Evolution and Ecology, University of California at Davis, Bodega Bay, CA 94923, USA

**Keywords:** metamorphosis, Echinoidea, hydrodynamics, shear forces, environmental cues, recruitment

## Abstract

Complex life cycles have evolved independently numerous times in marine animals as well as in disparate algae. Such life histories typically involve a dispersive immature stage followed by settlement and metamorphosis to an adult stage on the sea floor. One commonality among animals exhibiting transitions of this type is that their larvae pass through a ‘precompetent’ period in which they do not respond to localized settlement cues, before entering a ‘competent’ period, during which cues can induce settlement. Despite the widespread existence of these two phases, relatively little is known about how larvae transition between them. Moreover, recent studies have blurred the distinction between the phases by demonstrating that fluid turbulence can spark precocious activation of competence. Here, we further investigate this phenomenon by exploring how larval interactions with turbulence change across ontogeny, focusing on offspring of the sand dollar *Dendraster excentricus* (Eschscholtz). Our data indicate that larvae exhibit increased responsiveness to turbulence as they get older. We also demonstrate a likely cost to precocious competence: the resulting juveniles are smaller. Based upon these findings, we outline a new, testable conception of competence that has the potential to reshape our understanding of larval dispersal and connectivity among marine populations.

## 1. Background

### 1.1 Settlement, precompetence and competence in marine life cycles

A common pattern among a wide diversity of multicellular marine organisms—including vertebrates, invertebrates and some algae—is a dispersive life-history stage in the plankton followed by settlement to the sea floor [1–3]. In marine invertebrates, these dispersing stages are typically microscopic larvae, which undergo what can be a quite dramatic metamorphosis as they settle and begin the juvenile phase.

One feature that many taxa with this type of complex life history have in common (even across kingdoms) is that the transition to the sea floor at settlement is generally irreversible. In this sense, the ‘decision’ of where and when to settle is a crucial one, and larvae (and corresponding dispersive stages in non-animals) are predicted to use a range of environmental signals to help them accurately discern an appropriate place to settle [4]. These features can be chemical (e.g. compounds released by conspecifics or a favoured food source) or physical (e.g. the sound of waves breaking on the shore, light intensity or substratum roughness). Complicating matters, most dispersive organisms likely employ a combination of such signals [4–9].

A second feature typical of marine larvae is that they tend to develop through a *precompetent* period, during which they often feed and grow, but are thought to be incapable of settling and completing metamorphosis until they attain *competence* to settle under appropriate conditions [2,10]. Although the timing of the onset of the precompetent period tends not to be clearly defined, it is generally assumed that the selective advantage of the precompetent period is to ensure that the individual is sufficiently well developed to survive and thrive on the sea floor. For example, sea stars with feeding larvae can develop for a month or longer before attaining competence, at which time their adhesive structures mature, allowing a firm initial attachment at settlement [11]. By contrast (and curiously so), multicellular algal propagules are not known to divide and grow during dispersal, and this constraint may explain their non-existent or short precompetent periods [1].

### 1.2 Turbulence: a new class of settlement cue

Recently, we discovered that the trajectory of the precompetent period for larvae of the purple sea urchin (Echinodermata: Echinoidea: Strongylocentrotidae) *Strongylocentrotus purpuratus* (Stimpson) is not ‘pre-programmed’ as has been envisioned [12–15]. Instead, nominally precompetent urchin larvae (defined operationally by the absence of response to a strong settlement cue) will become competent to settle after a mere 3 min exposure to fluid turbulence [16]. Because turbulence levels under breaking waves on rocky shores differ from those in other ocean habitats [17], one implication of this finding is that turbulence might operate as a broad (metre to kilometre) ‘habitat-scale’ cue that gives larvae a general indication that they are in the right neighbourhood for settlement, with settlement itself only coming after larvae subsequently detect more localized cues (i.e. centimetre to millimetre scales) at the sea floor. Specifically, our results with purple urchins raise the possibility that larvae in the field respond to turbulent conditions that are diagnostic of a particular habitat type, such as exposed intertidal shoals versus calmer embayments. A second, potentially even more important implication of turbulence as an inducer of competence is that the precompetent/competent boundary is malleable, in that it can be modified by short-term environmental conditions.

An additional, accompanying issue is that larvae during the precompetent period may be forced to trade off some aspect of juvenile growth and/or survival against the likelihood of encountering suitable juvenile habitat (see [18,19] for a similar argument in competent larvae). This scenario may apply well to echinoderms in particular, as much of juvenile ontogeny is already underway during the precompetent larval period [20,21]. Thus, if an echinoderm larva waits to settle until the nominal competent stage, then it will presumably be able to complete more of juvenile development before settlement, increasing its likelihood of survival on the sea floor. On the other hand, if the larva waits and grows but misses the opportunity to reach good habitat it may forgo the chance to settle at all [22].

### 1.3 Turbulence-induced settlement and ontogeny

**Figure 1.**
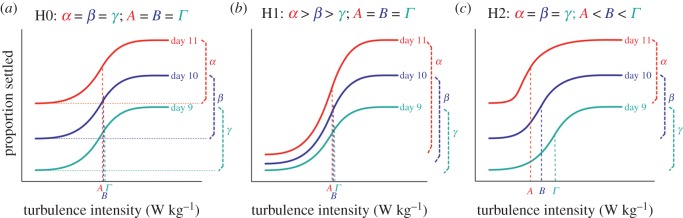
Schematic of null and alternative hypotheses for ontogenetic changes in the turbulence response. We here envision a series of hypotheses to account for changes in how larvae respond to turbulence as ontogeny proceeds. Each of the panels shows how the proportion of sand dollar larvae that settle following exposure to turbulence might change as a function of turbulence intensity, for three different larval ages (day 9 through day 11 after fertilization). From each of the dose–response curves shown, we can calculate the maximal proportion of larvae induced on that day (*lower case Greek letters*), as well as the turbulence intensity at which larvae become sensitive to turbulence exposure, represented by the inflection point of the curve (*upper case Greek letters*). See text for a full explanation of each hypothesis.

A scenario of selective costs and benefits as outlined above leads us to consider a series of hypotheses concerning how larval behaviour might change during the precompetent period in response to turbulence exposure (figure 1). These hypotheses are based on the conjecture that younger larvae could benefit from further time in the plankton, and thus might be more selective than more advanced larvae in their response to information about habitat quality. The following hypotheses (as well as the accompanying null hypothesis) pertain to how turbulence might induce competence in larvae as a function of their age:

H0: Larvae at a certain point during the precompetent stage begin to respond to turbulence, but the nature of this *response does not change* as the larvae continue to develop towards competence. In other words, any increases in settlement through ontogeny could arise simply because a greater fraction of larvae transition from precompetence to competence: there would be no difference between younger and older larvae in their reaction to turbulence (null hypothesis; figure 1*a*).
H1: Larvae react more strongly to a given stimulus as they get older. According to this hypothesis, an increasing proportion of precompetent larvae respond to turbulence and become competent to settle, indicating *increased responsiveness to turbulence* as ontogeny proceeds (figure 1*b*).
H2: Larvae respond to a lower level of stimulus as they get older. According to this hypothesis, older precompetent larvae respond to lower levels of turbulence than do younger precompetent larvae, indicating *increased sensitivity to turbulence* in older larvae, and increased selectivity of younger larvae to turbulence cues (figure 1*c*).

A corollary to these aforementioned scenarios concerns likely trade-offs associated with settling earlier in ontogeny. Specifically, we predict that precompetent larvae settling after exposure to turbulence do so at some cost relative to larvae that develop to nominal competence before settling.

Here we test each of these hypotheses and predictions in turn, using larvae of the Northeast Pacific sand dollar (Echinoidea: Clypeasteridae) *Dendraster excentricus* (Eschscholtz). We discuss the implications of our findings for life-history theory and for our understanding of the physiological ‘decision-making’ by larvae as they approach their competent period and subsequent commitment to settlement on the sea floor.

## 2. Material and methods

### 2.1 Experiment overview, definitions of terms and study species

**Figure 2.**
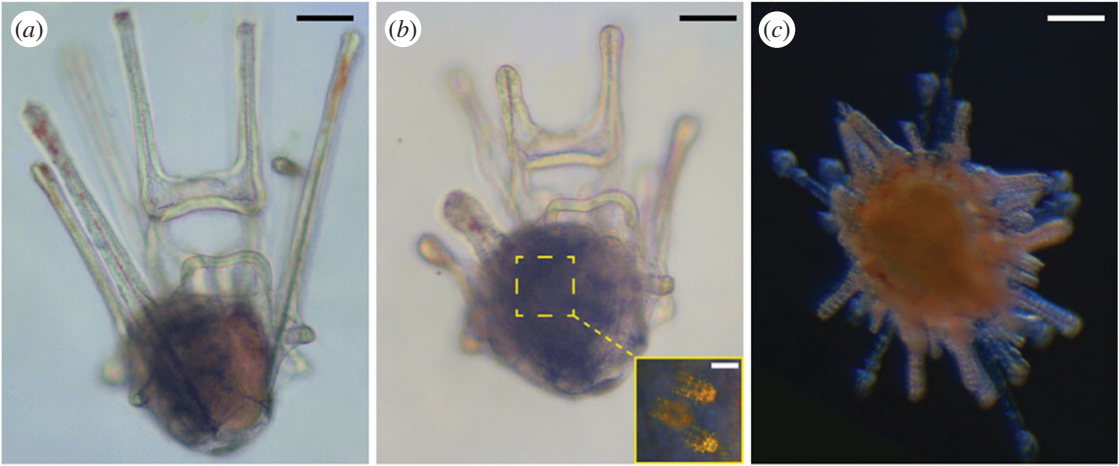
Representative precompetent and competent *D. excentricus* larvae. (*a*) Advanced precompetent larva, 11 days after fertilization (20–22°C). The rudiment is the dark area on the left side of the central body region; the stomach is the reddish area on the right side. (*b*) Early competent larva, 12 days after fertilization, at the same magnification as in (*a*). Note the shrunken larval arms, more rotund morphology, and the rudiment filling the entire central body region, all typical of competent larvae of these sand dollars. Inset shows a cross-polarized light magnified view of three developing adult-type spines within the rudiment of this larva. (*c*) Juvenile, at the same magnification as in (*a*) and (*b*), settled in response to 40 mM excess KCl (without turbulence exposure) on day 14, photographed on day 15. Scale bars: (*a*–*c*) 100 μm; (*b*) inset, 25 μm.

In our prior work [16], we examined turbulence-induced precocious competence in advanced precompetent larvae of the purple sea urchin. Here, we extend our taxonomic scope to focus on larvae of the sand dollar *D. excentricus*. *D. excentricus* adults live gregariously in intertidal or shallow subtidal beds throughout much of the Northeast Pacific, in both exposed and protected locations [23]. Their planktotrophic larvae have a very wide temperature tolerance range—from at least 8°C through 24°C [24] (J. Hodin 2014, unpublished data)—and when fed ad libitum at approximately 20–22°C, they develop synchronously and reach competence (figure 2) between 12 and 14 days after fertilization. Under these conditions, invagination of the echinus rudiment—the first stage of development of the incipient juvenile (comparable with soft tissue stage i in purple urchins [21])—occurs in 6-arm larvae between days 2 and 3, and the first calcification of juvenile structures within the rudiment occurs in 8-arm larvae on about day 6 (data not shown).

Here we provide definitions of four relevant terms regarding life cycle transitions in the ocean. We define *metamorphosis* as a more-or-less radical morphological transformation between two multicellular life stages [25]. In sea urchins, the morphological transformation (larva to juvenile) begins several days or even weeks before the habitat shift (planktonic to benthic). Specifically, juvenile structures in sea urchins begin to form over a protracted period within and on the left side of the functioning, feeding larva (e.g. [21]). Therefore, we refer to sea urchin metamorphosis as the entire period from the first formation of juvenile structures (‘rudiment’) in the larva until the juvenile begins to feed on the sea floor. The most dramatic event of metamorphosis is *settlement*: the (in most cases irreversible) transition that the larva makes from the planktonic to the benthic zone. We define sand dollar settlement in more precise terms for our experiments in §2.6. *Competence* is generally defined operationally as the stage at which a larva first becomes responsive to localized settlement cues; before this stage, the larva is thus defined as *precompetent*. We will provisionally adopt these latter two definitions for the purpose of the paper, though our data suggest reconsideration of these concepts; we provide new definitions in the Discussion.

Our overall approach was to rear larvae through their feeding larval stage using standard methods [24], and then, in the days preceding the competent stage, subject them to a brief turbulence exposure of a specified intensity in a Taylor–Couette cell (described further below). Immediately after exposure, we transferred the larvae into excess potassium chloride (KCl) in seawater for 1 h, which induces competent *D. excentricus* larvae to settle [26] (and can, based on our prior work, induce settlement in turbulence-exposed, precompetent purple sea urchin larvae [16]), and we then quantified settlement as it relates to turbulence intensity. In a subset of trials, we also used an extract of adult sand dollar bed sand as a natural inducer, to ensure that the results we detected were not limited to induction with KCl. Lastly, we examined one potential consequence of precocious settlement by comparing sizes of juveniles that resulted either from settlement of competent larvae or from precompetent larvae that settled early following exposure to turbulence.

### 2.2 Source populations and collection

We collected adult *D. excentricus* adults by snorkelling on 27 May 2014 to a subtidal population (approx. 100 m offshore and 1.5 m below the surface at mean lower low water) approximately 30 m east of Municipal Pier 2 in downtown Monterey, CA, USA. We transported the sand dollars to Hopkins Marine Station (‘HMS’; Pacific Grove, CA, USA) and maintained them in sand in running seawater aquaria until spawning.

### 2.3 Larval culture

We obtained gametes by intracoelomic injection of (depending on the size of the adult) between 0.2 and 0.5 ml 0.55 M KCl in distilled water, and fertilized by standard methods [24]. For each larval culture, we combined approximately equal numbers of embryos from three crosses between one male and each of three females, with each combination resulting in greater than 90% fertilization success. We set up larval cultures in this way on three consecutive days (with different sets of adults each day) on 27–29 May 2014, and then again (with different sets of adults) on 22 August 2014.

In the latter spawning, we transferred a large portion of the unfertilized eggs from each female into individual 250 ml culture flasks containing 0.1 g l^−1^ ampicillin in 0.45 μm Millipore-filtered natural seawater (MFSW) with UV treatment. Storing eggs in this manner at ambient temperature (here 14°C) renders them fertilizable for a week or longer [27,28]. We stored sperm dry at 4°C. Then, on 24 and 25 August, we set up fertilizations as above from this same batch of sperm and stored eggs, following several washes in MFSW to remove any residual antibiotic. Thus, for the September 2014 experiments, we raised three batches of genetically comparable larvae of different ages in parallel.

We began feeding 4-arm plutei 1 day after fertilization. Initial larval densities were 0.5–1 larva ml^−1^, which we lowered to 0.2–0.3 larva ml^−1^ on day 5 or 6 (8-arm larvae; early rudiment stages). We fed larvae 5000 cells ml^−1^ *Rhodomonas* spp. For the June 2014 experiments, we transported embryos and larvae on 29 May from HMS to Bodega Marine Laboratory (‘BML’; Bodega Bay, CA, USA), where we maintained them as above until experimental trials. For the September 2014 experiments, we similarly transported larvae on 27 August from HMS to BML.

At both HMS and BML, we cultured embryos and larvae in 0.45 μm MFSW with UV treatment, in gallon jars with mechanical stirring [24] at room temperature, which varied from 20 to 22°C.

### 2.4 Generating turbulence in the laboratory

To generate turbulence intensities (quantified in terms of the energy dissipation rate, in units of W kg^−1^) ranging from those found in open ocean waters to those arising on wave-battered coasts, we employed a Taylor–Couette cell [29], an apparatus composed of two vertically oriented, coaxial cylinders separated by a 3.5 mm gap that contains seawater (described in greater detail in [16]). We held the stationary inner cylinder, and thus the water in the gap, at 19–21°C by means of a circulating water stream from a temperature-controlled water bath passing through the cylinder's interior. During operation, the outer cylinder rotated at a prescribed speed causing relative motion between the cylinders and thereby shearing the seawater between them. At rotation speeds employed for testing sand dollar larvae, the sheared flow was turbulent [16].

### 2.5 Larval exposure to turbulent shear

In early June (batches A and B) and early September 2014 (batch C) at BML, we exposed sand dollar larvae to prescribed levels of turbulence, using larvae that were between 7 and 14 days old. We first concentrated larvae by reverse filtration and selected 25–35 larvae into individual 125 ml glass beakers at a density of one larva per 3–4 ml MFSW. Larvae in these experiments were developing synchronously; we only eliminated larvae from consideration if they were either obviously delayed in development (less than 5% of the larvae; data not shown), or in the case of late-precompetent larvae, if they adhered to glassware at the time of selection or during transfer into exposure treatments. Otherwise, we chose larvae haphazardly. Then, we randomly assigned beakers to treatment and replicates, and began the exposures.

For each treatment run, we gently poured the entire contents of a 125 ml beaker into a finger bowl, and used a glass Pasteur pipette to introduce all of the larvae into 150 ml of MFSW within the Taylor–Couette cell. We then subjected the entire water volume within the apparatus to a specified level of turbulent shear for 3 min. Immediately following each treatment run, we gently poured the larvae within the Taylor–Couette cell into a 1 l glass beaker already containing approximately 100 ml of MSFW (to minimize additional stimulus to larvae during the pour), and we rinsed the Taylor–Couette cell twice with 19–21°C MFSW to capture any remaining larvae. We generally recovered greater than 90% of the larvae, all of which we used in ensuing settlement assays. We then rinsed the Taylor–Couette cell thoroughly with distilled water to ensure that no living larvae were transferred to subsequent trials, and we initiated the next trial.

In concert with the treatment exposures, we exposed randomly selected batches of larvae to one of two control conditions. For larvae assigned to ‘unmanipulated controls’, we poured the contents of the 125 ml beakers into finger bowls, as described above, and then immediately subjected them to settlement assays. For larvae assigned to ‘handling controls’ (0 W kg^−1^ (= 0 r.p.m.) treatments), we treated them the same as larvae assigned to the various turbulence exposure treatments, except we did not activate the Taylor–Couette cell during the 3 min that larvae were within it, thereby controlling for manipulations associated with transfer of larvae into and out of the apparatus.

### 2.6 Quantification of settlement

Following turbulence treatments, we assessed settlement by exposing all larvae from a run to 8 ml of 40 mM excess KCl in MFSW for 1 h at 19–21°C in a single well of a non-tissue culture-treated 6-well plate, followed by recovery in 8 ml MFSW, as in previously employed protocols [16,26,30]. We scored a larva as settled after this 1 h exposure if skin had begun to withdraw from the tips of the larval skeletal rods, and we verified continued withdrawal of skin over the next several hours.

To confirm our scoring of settlement in the KCl exposures, and to ensure that the settled larvae would complete metamorphosis normally, we re-examined all settled larvae following an additional 12–24 h of recovery in MFSW at 19–21°C. This evaluation verified that greater than 95% of all larvae had been scored correctly the previous day, with adoption by 12–24 h of the definitive juvenile morphology, with emergent and active tube feet and spines. We detected no post-settlement mortality. Larvae from all treatments that we had scored as not settled also recovered normally, were swimming within 1 h of recovery, and remained swimming after 24 h with no apparent ill effects of the treatment.

### 2.7 Natural inducer experiments

In one set of trials examining whether turbulence effects were associated exclusively with KCl induction or would manifest more broadly (e.g. with a natural inducer), we used an extract of surface sand that we collected in August 2014 from a high intertidal sand dollar bed in Alki Beach, Seattle, WA, USA (approx. 1.5 vertical feet above the mean lower low water line), and which we then froze at −20°C in 50 ml aliquots the same day. Our unpublished observations suggest that sand collected from intertidal sand dollar beds of *D. excentricus* (as found in WA) is a more potent settlement inducer than sand from subtidal populations (as found in CA), regardless of the geographical source of sand dollar larvae tested.

To prepare the sand cue, we thawed 25 ml of the Alki sand, shook it vigorously with 25 ml of MFSW for 30 s, allowed the large sand particulates to settle out for 10 s, and then decanted off the opaque supernatant into a new tube. We then diluted this supernatant 1 : 1 with MFSW, resulting in what we termed a 100% sand extract. We used this extract for settlement tests at various dilutions in MFSW within a few hours of thawing. We conducted sand extract exposure trials in 6-well plates in which the remaining sediment suspended in the sand extract had been allowed to settle out for at least 30 min, rendering the water above reasonably transparent.

For our natural inducer trials, we transferred 25 larvae into the Taylor–Couette cell, which we activated at approximately 6 W kg^−1^ (500 r.p.m.) for 3 min. Upon recovery of larvae from the Taylor–Couette cell, we immediately exposed them for 60 min to 8 ml of a 50% sand extract in a single well of a 6-well plate, which we then diluted to 35% strength (by the addition of 3.5 ml MFSW) for eight additional hours to allow for the completion of settlement in partially settled larvae, while limiting de novo settlement during this protracted exposure period. After the 9 h total exposure, we scored a larva as ‘settled’ only if its skin was fully withdrawn from its larval arms with juvenile rudiment everted. We compared three replicates of these 6 W kg^−1^-exposed larvae to three replicates of unmanipulated control larvae, treated in parallel as above.

### 2.8 Juvenile size

In early September at BML, we gathered all juveniles that had settled in 40 mM excess KCl following 3 min, Taylor–Couette cell exposure trials at approximately 2 W kg^−1^ (290 r.p.m.) on day 11 (24 August fertilization) and photographed them after 24 h at 20× with a Leica (Solms, Germany) MC170 HD camera mounted on a Leica M125 dissecting microscope, using Leica Application Suite software (v. 4.5). We did the same for larvae that had settled following 1 h of 40 mM excess KCl exposure on day 14 (i.e. nominally competent larvae that had not been spun in the Taylor–Couette cell; 22 August fertilization). We measured the test diameters in two orthogonal dimensions of these juveniles from the photos using ImageJ (v. 1.49D). We calculated the test area for each juvenile, approximating each test as an ellipse in cross section. We captured additional images of larvae (figure 2) using the same set-up mounted on a Leica DM1000 compound microscope.

### 2.9 Statistical analyses

For the ontogeny comparisons from days 7 to 12 at 6 W kg^−1^ (500 r.p.m.) versus unmanipulated controls, we used an ANCOVA, with developmental day as a covariate, and a Tukey's HSD test for *post hoc* comparisons. We employed a single batch of larvae (batch A) for this experiment.

Using the dose–response data on our three larval batches (‘A’, ‘B’ and ‘C’), we evaluated the hypotheses outlined in figure 1 as follows.

To quantify potential shifts in larval ‘responsiveness’ (H1, figure 1*b*), we determined the maximum (max.) and minimum (min.) proportion of larvae that settled in response to exposure to KCl following any turbulence treatment (including handling controls: 0 W kg^−1^, 0 r.p.m.) within each replicate dose response from days 9–11 (*N*=15). We considered this minimum proportion as the background settlement rate (i.e. the proportion of larvae that were competent) within that set. To calculate the maximum proportion of larvae that became competent and settled in response to turbulence, we performed the following calculation for each replicate dose response:

$$\frac{\text{max.}-\text{min.}}{1-\text{min.}}.$$

The resulting value represents the fraction of formally precompetent larvae (i.e. the quantity (1 − min.)) that became competent to settle as a consequence of turbulence exposure. Then, to assess statistical confidence in the relationship between maximum proportion of larvae that became competent to settle in response to turbulence and developmental day (9–11), we used a mixed linear model with batch (A–C) as a random effect. In the model, we allowed batch effects to vary both the intercept and slope of the regression, and used an unpaired *t*-test (with Satterthwaite approximations to degrees of freedom) to assess whether developmental day is a significant predictor of the regression.

To quantify potential shifts in ‘sensitivity’ (H2, figure 1*c*), we used a generalized mixed linear model to compare the larval response to turbulence on days 9 and 11. We began with a basic logistic model including only turbulence intensity and developmental day (Akaike information criterion (AIC) score: 542.3), and then subsequently added the following additional variables in turn to arrive at the best-supported model based on AIC score [31]: random effects (batch and runs nested within batch; AIC score: 481.8), and a quadratic term for turbulence intensity (to approximate the apparent drop off at high turbulence intensities; AIC score: 465.7). The model failed to improve further when we added either an additional variable for the interaction between turbulence intensity and age (AIC score: 467.1), or for an interaction between the quadratic term and age (AIC score: 466.7). As expected, the model also failed to improve with inclusion of both of these interaction terms (AIC score: 468.3).

In the best-supported model, the quadratic variable was also highly significant (*p*<0.001). Therefore, for further analysis, we chose this latter model, which included the following variables: turbulence intensity, developmental day, the quadratic term for turbulence intensity and random effects.

Using this model, we generated best curve fits, producing mean response curves on days 9 and 11 with ±95% confidence intervals (CIs). To determine if these inflection point estimates on days 9 and 11 were statistically different, we ran a non-parametric bootstrap algorithm (with random effects as above) to generate 10 000 inflection point estimates. We then calculated the 95% confidence range for these inflection point estimates on days 9 and 11 using the boot.ci function in R. This same function allowed us to identify the maximum confidence range (*X*) of these inflection point estimates on days 9 versus 11 that showed no overlap. We report the *p* value for these inflection point estimates as 1−*X*.

For the natural inducer data, we used unpaired *t*-tests to test for differences between turbulence-exposed larvae (6 W kg^−1^ (500 r.p.m.)) and unmanipulated controls. We did the same for the corresponding KCl induction data.

For the juvenile size data, we compared the mean test areas of day 12 juveniles (with turbulence exposure at 2 W kg^−1^ (290 r.p.m.)) with the day 15 juveniles (without exposure). Because we did not replicate the turbulence exposures or post-settlement recovery conditions, we tested for bias in the data by performing 10 000 comparisons between sets of 10 randomly chosen juveniles from each treatment, all 10 000 of which had *p*<0.005 (not shown).

For all data, we assessed normality using *q*–*q* plots. We performed all statistical analyses using R (v. 3.1.0).

## 3. Results

### 3.1 Ontogenetic scope of the response to turbulence

**Figure 3.**
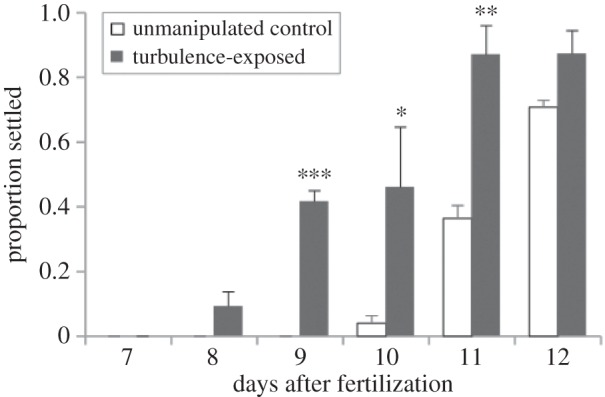
Turbulence exposure in sand dollars shortened the time to competence by approximately 20%. On each day indicated, we subjected three replicates of 20–25 larvae each to 3 min of turbulence exposure at approximately 6 W kg^−1^ (500 r.p.m.), and then immediately transferred them into 40 mM excess KCl in MFSW; we also transferred three replicates of 20–25 larvae directly into 40 mM excess KCl in MFSW (i.e. with no turbulence exposure; unmanipulated controls). All larvae in this analysis were from the same batch (larval batch A, fertilized 27 May 2014). Error bars are s.e.m. Asterisks indicate significant differences on the day indicated between turbulence-exposed and unmanipulated control larvae (*p<0.05; **p<0.01; ***p< 0.005), as determined by two-way ANOVA (*F*_1,24_=47.36; *p*<0.001) and Tukey's HSD *post hoc* comparisons.

Sand dollar larvae at days 7–12 after fertilization settled approximately 2 days earlier and at higher rates overall if we first exposed them to intense turbulence in a Taylor–Couette cell (figure 3; ANCOVA, *F*_1,10_=31.25, *p*<0.001). As in purple sea urchins [16], turbulence exposure does not itself induce settlement in sand dollars (data not shown). Instead, larvae exposed to turbulence must be presented with a localized cue (e.g. excess potassium chloride in seawater or a natural inducer) before they settle and transform to the juvenile stage.

The data in figure 3 thus demonstrate an increase in turbulence-induced settlement as ontogeny proceeds, but this outcome could emerge in several ways (figure 1); our analysis of the dose response data, as explained above and detailed below, allows us to distinguish among these different possibilities.

### 3.2 Changes in turbulence-induced settlement through the precompetent period

**Figure 4.**
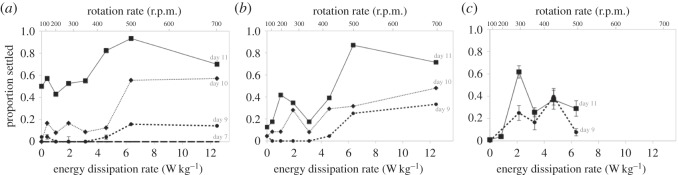
Sand dollar larvae show substantial batch-to-batch variation in the turbulence response across ontogeny. (*a*) Larval batch A, fertilized 27 May 2014. (*b*) Larval batch B, fertilized 28 May 2014. Each of the data points in (*a*) and (*b*) are results from single runs of 25 larvae, with the exception of the day 9 batch A data, which we replicated once (data points on day 9 in (*a*) show the mean of the two runs; error bars are s.e.m). (*c*) Larval batch C, fertilized 22 August 2014. Each of these data points are means of four runs at each speed with 20–25 larvae each; error bars are s.e.m. Note that in (*a*–*c*), we do not indicate the error along the *x*-axis in each of the Taylor–Couette cell rotation rates that we employed, which we estimate to be approximately ±25 r.p.m. Each graph shows the energy dissipation rates (in W kg^−1^) on the lower *x*-axis, and the corresponding rotation rates (spin speeds in r.p.m.) along the upper *x*-axis. We only tested day 7 larvae from batch A and day 10 larvae from batches A and B.

We reared several independent batches of larvae until they were between 7 and 11 days old, and then exposed them to turbulence intensities spanning conditions characteristic of quiescent waters, to calmer offshore flows, to violently breaking waves (i.e. turbulent energy dissipation rates from 0 to in excess of 10 W kg^−1^ (see [16,17])). Owing to substantial variation among batches in how larvae responded to turbulence across ontogeny (figure 4), we undertook a series of analyses to test H1 and H2 (figure 1) using our data from the three batches considered together, as we detail in the following sections.

#### 3.2.1 Shifts in larval responsiveness to turbulence (H1)

**Figure 5.**
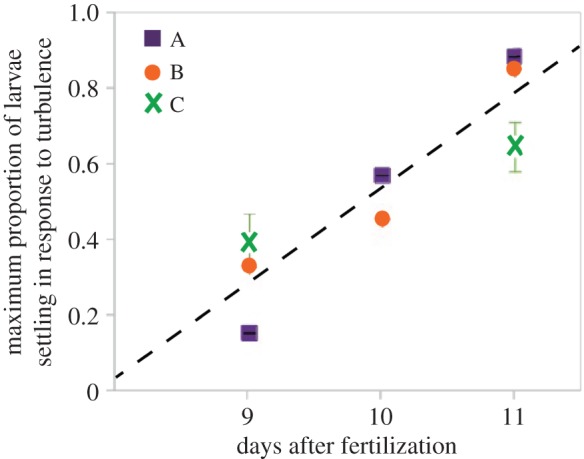
Precompetent sand dollar larvae show increasing responsiveness to turbulence as ontogeny proceeds. Shown are the maximal proportion of larvae in each of our three batches (A, B and C; figure 4) that settled on a given day with the background level of settlement in that batch subtracted out (thus excluding larvae that were nominally competent); error bars are s.e.m. (note that the data from batch A days 10 and 11 and batch B days 9, 10 and 11 were unreplicated, thus show no error bars). This analysis reveals a marked and steady increase in the proportion of nominally precompetent larvae that responded to turbulence as ontogeny proceeds. The estimated slope (±s.e.m.) for the regression is 0.24 (±0.07) maximum proportion settled per age (unpaired *t*-test: *t*_7.9_=3.25, *p*<0.02), and the estimated intercept (±s.e.m.) is −1.90 (±0.74; *t*_7.9_=−2.56, *p*<0.04).

When we used these data to test H1, our results suggest that larvae indeed exhibited increased ‘responsiveness’ to turbulence exposure as ontogeny proceeds (figure 1*b*). In particular, we see an increase as larvae get older in the peak proportion of nominally precompetent larvae that nevertheless settled across all turbulence intensities when subsequently given a settlement inducer (figure 5). In our analysis of these data, age of larvae was a significant predictor of the regression (*t*-test; *t*_7.9_=3.251; *p*<0.02), thus supporting H1.

#### 3.2.2 Shifts in larval sensitivity to turbulence (H2)

**Figure 6.**
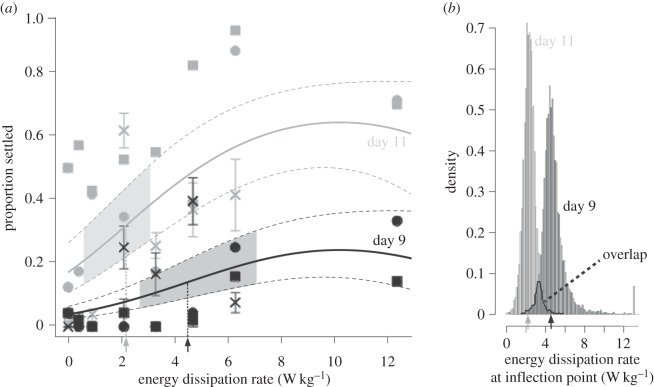
Precompetent sand dollar larvae show evidence of increasing sensitivity to turbulence as ontogeny proceeds. (*a*) Shown are the best-fit curves (solid curves) ±95% CIs (dashed curves) generated by our best-supported general mixed linear model (see Material and methods for details and comparisons with other models). The *lines* and *symbols* (batch A, squares; batch B, circles; batch C, crosses) show the data from day 9 (black) and day 11 (grey). Error bars are s.e.m (note that the data from batch A day 11 and batch B days 9 and 11 were unreplicated, thus show no error bars). The inflection points of the best-fit curves for day 9 (black arrow) and day 11 (grey arrow) are shown along the *x*-axis and indicated by the black and grey vertical dotted lines, respectively. The *shaded areas* within the day 9 and day 11 CI curves indicate the range of 95% CIs in our respective inflection point estimates based upon 10 000 non-parametric bootstrap samples. (*b*) The range of inflection point estimates (and 8.5% overlap) from these bootstrap samples on days 9 and 11. *Arrows* as in (*a*). Note that the *y*-axis units of density are linearly related to the proportion of bootstrap samples showing a given range of inflection point estimates.

Our next analysis tested whether larvae became more sensitive to a given level of turbulence as ontogeny proceeds (H2; see figure 1*c*), by examining the position of inflection points. For this analysis, we fit the combined settlement data for days 9 and 11 (i.e. figure 4*a*–*c*) to a logistic curve using a mixed linear model (figure 6*a*). The model with the most support was one with a quadratic term included to account for the apparent decline in settlement seen at higher turbulence intensities in the majority of runs shown in figure 4 (see Material and methods for details on and AIC scores of the various model combinations). In the logistic-quadratic regression, the slope (±s.e.m.) was 0.43 (±0.06; *p*<0.001) log odds of settling per unit increase of W kg^−1^, the quadratic term was −0.02 (±0.00; *p*<0.001) log odds of settling per squared unit increase of W kg^−1^, and the intercepts (±s.e.m.) were as follows: day 9, −3.35 (±0.30; *p*<0.001); day 11, −1.61 (±0.37; *p*<0.001) log odds of settling.

Using this best-supported model, we generated best-fit curves ±95% CIs for the data from days 9 and 11, and from a non-parametric bootstrap of the data, calculated and compared the inflection points (figure 6). The 95% CIs on the inflection point for the response on day 11 (mean: 2.2 W kg^−1^; 95% confidence range: 0.7–3.1 W kg^−1^) showed slight overlap with the range on day 9 (mean: 4.5 W kg^−1^; 95% confidence range: 2.7–7.1 W kg^−1^), as seen graphically in figure 6*b*. These data indicate a trend (*p*=0.085) in which nominally precompetent larvae become more sensitive to turbulence as ontogeny proceeds, thus lending borderline support to H2.

### 3.3 Natural inducer

**Figure 7.**
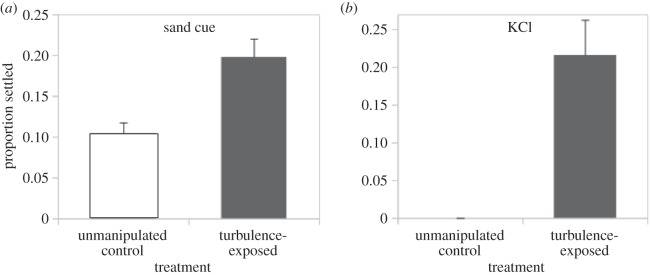
Precompetent sand dollar larvae will settle following turbulence exposure in the presence of a natural inducer. (*a*) Twice as many larvae settled in sand extract following a 3 min exposure to intense turbulence (approx. 6 W kg^−1^; unpaired *t*-test, *t*_4_=−3.72, *p*=0.02). (*b*) Settlement in 40 mM excess KCl with this same batch of larvae also showed approximately 20% settlement following the same level of turbulence exposure, and was elevated relative to a no-turbulence, unmanipulated control treatment (Kruskal–Wallis, H2 =−9.39, *p*<0.01), as well as an accompanying handling control (not shown). Note that we conducted this experiment on day 12 from our 25 August 2014 fertilization, a batch of larvae that was delayed relative to our other batches due, we suspect, to colder room temperatures and slightly more crowded conditions. These day 12 larvae were approximately the same stage (using our staging scheme modified from [20]) as the day 9–10 larvae from our other fertilizations (data not shown). Error bars are s.e.m.

In addition to the aforementioned results based on the use of a chemical settlement inducer (40 mM excess KCl), we also observed effects of turbulence on settlement when we used the natural inducer derived from surficial sand from a *D. excentricus* sand dollar bed. In particular, at turbulence intensities that caused 20% of larvae to settle with KCl induction (approx. 6 W kg^−1^; again using a 3 min exposure), a similar proportion of larvae settled in response to sand cue (figure 7). As is often the case (e.g. [32]), the background settlement levels differed here between KCl and natural inducer.

### 3.4 Cost of settling early: juvenile size

**Figure 8.**
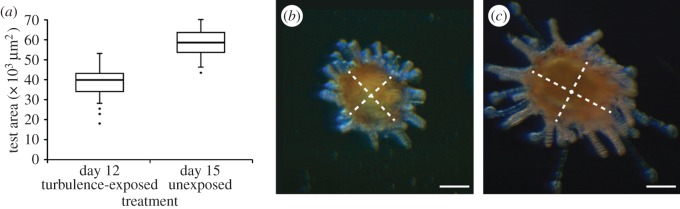
Precompetent larvae primed by turbulence exposure settle at a smaller size. (*a*) Mean test area in 12-day-old juveniles (*n*=49) deriving from turbulence exposure (approx. 2 W kg^−1^ for 3 min) while precompetent, compared to 15-day-old competent juveniles (*n*=29) never exposed to turbulence. Shown are standard Tukey box plots with the dots as outliers. Representative 12-day-old turbulence-exposed juvenile (*b*) and 15-day-old control juvenile (*c*) from this experiment, photographed at the same magnification. White dotted lines indicate the major and minor axes of the ellipses used to estimate cross-sectional test area. Note also the relatively shorter spines in (*b*), another indication of the precocious state of these turbulence-induced juveniles. Scale bars, 100 μm. The effect size here (mean size on day 15 − mean size on day 12) is 19 437 μm^2^.

Although our cultures were characterized by quite synchronous development, it is theoretically possible that the subset of larvae that settled precociously in response to turbulence comprised a slightly larger size class built from the upper tail of the overall size distribution of precompetent larvae. If so, it is conceivable that turbulence-exposed, precompetent larvae might show similar sizes at settlement when compared with older, formally competent larvae that settled in the absence of turbulence. Our data, however, exhibit no support for this scenario. Juveniles that derived from turbulence-exposed precompetent larvae had significantly smaller tests than did juveniles that derived from fully competent larvae that settled without turbulence 3 days later (figure 8; *t*-test: *t*_77_=−1.50, *p*<0.001). For reasons of logistics we were not able to track these juveniles much beyond settlement, but other studies have shown strong consequences of reduced size at metamorphosis for early post-settlement survival (e.g. [33]).

## 4. Discussion

In prior work we demonstrated how brief exposures to strong fluid turbulence can shift the timing of the precompetent/competent transition in echinoderm larvae [16]. Here we offer additional insights into how such a response may change across ontogeny. In particular, our experiments reveal that older larvae exhibit heightened responsiveness and a trend towards greater sensitivity to turbulence exposure as they approach the nominal competence stage, and we demonstrate a likely cost to precocious, turbulence-induced settlement. These findings point to testable predictions regarding selective trade-offs associated with a larva abandoning its planktonic existence and settling on the sea floor.

### 4.1 Turbulence responses in the precompetent period

Echinoderm larvae in general seem well adapted to make the dramatic transition from their planktonic stage to the sea floor at settlement. About mid-way through the larval development period in those species that feed—which can be weeks to months or longer depending on species, temperature, food and other factors—the pentameral juvenile structures begin to grow and differentiate inside and on the left side of the otherwise bilaterally symmetric larva (reviewed in [34–36]). The latter half of larval development is dominated by the formation of a functional juvenile on this left side of the still-feeding larva. During the precompetent stage, the larva is considered incapable of completing metamorphosis and settling even if given an appropriate and potent settlement inducer. Then, at a certain stage, the larva more-or-less suddenly becomes responsive to those same inductive cues, and hence is operationally defined as being competent [2].

Particularly in echinoids (sea urchins, sand dollars and their kin), the distinction between precompetent and competent larvae under placid flow conditions in the laboratory has been demonstrated in numerous species. Such observations are facilitated by the ease with which many such species can be reared synchronously, and by the availability of both specific (such as algal extracts) and general (such as elevated KCl) settlement inducers in many taxa [26,30,37–42]. Thus, our discovery that this more-or-less discrete precompetent/competent boundary can be sensitive to turbulence was unexpected [16].

Our finding here of an approximate 20% shortening of larval development to competence in sand dollar larvae following turbulence induction is substantial. If extrapolated to the life-history trajectory of a more typical temperate, planktotrophic echinoderm larva with a one- to two-month larval period, this advancement of competence would represent a shortening of the larval period by one to two weeks [21]. Given the prominence assigned to the length of the larval period in ecological models of the life histories of marine organisms (e.g. [19,43–50]), and its function in determining patterns of dispersal (e.g. [51,52]), the broader implications of this turbulence-induced shortening of larval development may be considerable.

### 4.2 Changing responsiveness and sensitivity to turbulence during the precompetent period

Our experiments were designed to parallel what might happen when precompetent larvae approach the nearshore zone, where they would begin to experience substantial increases in turbulence intensity, ranging from gentler conditions to those only observed under large breaking waves in the surf zone [16,17,53,54]. We set out to test whether larvae of different ages would differ in their response to this increase in turbulence intensity, and in a manner that might indicate changes in the selective regime underlying the settlement response across ontogeny.

We envisioned three possible responses of larvae of different ages to turbulence (figure 1) which we introduced as a null hypothesis (H0) and two alternative (but not mutually exclusive) hypotheses (H1 and H2). Briefly, H1 predicted that larvae would react more strongly to a given stimulus as they get older (increased responsiveness with ontogeny; figure 1*b*), H2 predicted that larvae are activated by decreasing levels of stimulus as they get older (increased sensitivity with ontogeny; figure 1*c*), and the null hypothesis (H0) predicted that increases in settlement through ontogeny could arise simply because a greater fraction of larvae transition from precompetence to competence, with no change in the nature of the response over time (figure 1*a*).

Our analysis of the scope of the maximal turbulence effect across ontogeny (figure 5) supports H1, indicating that older, nominally precompetent larvae are indeed more responsive to turbulence than are younger larvae. With regard to H2, the most striking pattern that we observed was significant batch-to-batch variation in the response of larvae of matched ages to our tested range of turbulence intensities (figures 4 and 6). We observed this pattern despite the fact that all of our adults came from the same population, and all of our larval batches were mixed female (half-sib) fertilization designs. As such, we cannot currently determine the relative contributions of genetic and environmental factors and their interactions in explaining the observed variation. This scope of variation is consistent with the ‘sweepstakes reproductive success’ hypothesis (reviewed in [55]), which predicts that variation within populations of broadcast spawners in the ocean results in larvae that differ widely in their adaptation to varying oceanic conditions in the hope of a rare ‘sweepstakes-winning’ match.

With the caveat related to batch-to-batch variation in mind, we analysed our replicate data among and within batches to attempt to identify any evidence for shifts in larval sensitivity to turbulence exposure through ontogeny (H2; figure 1*c*). Of all of the models that we examined, the one that best accounted for the data (figure 6) indicated a substantial shift in the mean inflection point of the best-fit curves (i.e. the point at which the cohort of larvae responded most rapidly to increasing turbulence) from 4.5 W kg^−1^ on day 9 to 2.2 W kg^−1^ on day 11. A large majority (91.5%) of our bootstrap runs of the model parameters continued to show a separation between these inflection point estimates on days 9 and 11 (i.e. *p*=0.085), lending qualified support to H2.

We caution that we cannot be certain that the combined logistic-quadratic model that we chose is truly the best possible one to describe these data. For this reason, as well as our borderline *p*-value and observation of substantial batch-to-batch variation in the sensitivity of larvae that we mentioned previously, further experiments are necessary to demonstrate a definitive and ecologically meaningful shift in sensitivity to turbulence. Nevertheless, we note that the turbulence levels cited above are within the range expected for nearshore conditions on the west coast of North America [16,17,53,54], where *D. excentricus* is found. This correlation suggests that the responses could be ecologically relevant, and thus that larvae could use turbulence intensity as an environmental signpost of their arrival to the neighbourhood of potentially suitable habitat. The substantial spatial dimensions over which turbulence intensities vary (metre to kilometre) may provide an effective ‘habitat-scale’ indicator distinguishing (for example) between quiescent embayments and exposed rocky coasts. This type of information contrasts with that provided by chemical or other recognized cues, which usually operate over much smaller spatial dimensions (millimetre to centimetre) and provide clues for larvae about very localized features of habitat.

Taken together, our data support H1 and possibly H2, and strongly suggest that marine larvae have the ability to adjust their turbulence responses during ontogeny: in other words, the larval response to turbulence is not an all-or-none response, and is thus an example of phenotypic plasticity. Furthermore, these findings raise the exciting prospect of different populations and species tuning their response to turbulence depending on adult habitat: an example of natural selection. Finally, our observations of ontogenetic shifts in larval responsiveness (H1) and sensitivity (H2) to turbulence are consistent with a scenario of cost–benefit trade-offs, indicating that larvae may become less choosy as they proceed towards nominal competence.

### 4.3 Cost to settling early

The most obvious potential cost to an echinoid in settling early is that its juvenile structures would be under-developed [21,56,57]. Settling early would thus have expected costs in terms of juvenile growth, competition and/or protection from predation (e.g. [58,59]). We observed that juveniles resulting from turbulence exposure while precompetent were more than 30% smaller than their counterparts who had settled when they had reached competence in the absence of turbulence exposure, three days later. Although these data are strongly suggestive of a cost to settling early, a definitive demonstration of the nature of this cost would require rearing of these juveniles under a variety of conditions, and would be an important extension of this work.

Still, the dramatic difference in juvenile size that we observed, coupled with our evidence for a shift in turbulence responsiveness during ontogeny, suggests a scenario where larvae are balancing the likelihood of settling in an appropriate habitat with the chance of surviving there once they definitively settle. In younger stages, the chance of survival may be sufficiently low that larvae use stricter criteria to determine if they are in suitable habitat for settlement. By contrast, in older larvae, their higher potential for survival on the sea floor—coupled with the combined likelihood of predation in the plankton and advection away from the nearshore—may result in a selective relaxation of the turbulence conditions sufficient to activate competence (see [60] for an analogous analysis regarding so-called ‘desperate larvae’).

### 4.4 Rethinking competence

Much attention has been given to the variation in the potential length of the competent period in marine larvae, with numerous studies describing how long a larva can remain competent in the absence of an inducer (reviewed in [61]). In sum, the length of the competent period can vary substantially, both within and among taxa, and even within a single brood. At one extreme of the evolutionary continuum are generally short-lived, ‘desperate’ larvae that will become less choosy with respect to an appropriate settlement site as the competent period proceeds [42,62–65]. Such larvae will ultimately settle ‘spontaneously’ if no cue ever becomes available. At the other extreme are larvae that would rather meet ‘death before dishonour’ [61]; larvae of such species deprived of an appropriate settlement cue will remain competent and swimming until they die from lack of energy.

Less attention has been given to a second feature regarding the timing of competence: namely, when the competent period begins, and what controls its onset. The common observation across marine invertebrates (and even marine fish and some algae [66–69]) is that dispersal stages pass through an obligate precompetent period during which they are incapable of responding to cues and settling to the sea floor. The transition in marine invertebrate larvae from precompetence to competence has been described as being ‘pre-programmed’ [12–15], in the sense that there is a certain degree of growth and/or differentiation that must occur before the larva is even capable of undergoing settlement. In this conception, the attainment of competence can be considered a strictly developmental phenomenon, initiated either by sufficient development (within larvae) of structures needed by the juvenile to function on the sea floor [2], or by the maturation of a sensory system capable of receiving environmental settlement cues (e.g. [70–72]), or both. The justification for this ‘developmental’ view is an eminently reasonable one, as larvae must develop in the plankton until the stage at which they can successfully transition to the sea floor. In this sense, the precompetent period may represent both a developmental as well as a selective constraint.

Nevertheless, several sets of observations in the literature imply that a more nuanced view of the onset of competence may be warranted. First, in almost every case examined, authors have been unable to define a reliable morphological indicator of competence (e.g. [14,15,73,74]; J. Hodin 2014, unpublished data; but see [75]). Second, several studies have indicated that the timing of the onset of competence can differ substantially depending on the cue (e.g. [30,76–78]). Third, it is commonly observed that larvae from different batches (and even within a batch) can become competent at differing ages, even when raised under similar conditions (e.g. [13,74,79–81]), suggesting within-population genetic variation in the propensity to settle [82].

Our findings reported here and previously [16] add an additional layer of complexity. Turbulence does not induce settlement in echinoid larvae directly; instead, turbulence primes larvae to respond to a localized settlement inducer. Indeed, our results may explain why agitation has been previously observed to heighten settlement responses of some annelid and mollusc larvae [67,79,83–85]. Furthermore, our two-step conception of turbulence as a habitat-scale cue, priming larvae to respond to more localized cues, is also consistent with the analogous proposal of Trapido-Rosenthal & Morse [86]: increases in diamino acids from dissolved organic matter might indicate to red abalone larvae that they have arrived into nutrient-rich coastal regions where more localized cues could be employed.

From all of the above, we here propose a modified view of a larva's approach to competence, dividing larval development into three phases:

— *Phase 1: immature larva*. At this stage, the larva is not sufficiently well developed to successfully complete settlement under any circumstance, whether appropriate environmental cues are present, or not. This situation can derive from an insufficiently well-developed juvenile anlage (as in echinoderms), or the lack of a developed sensory system to respond to settlement cues (as in some molluscs; e.g. [71,72]), or both.
— *Phase 2: precompetent larva*. At this stage, the larva has the ontogenetic capacity to settle, but does not do so if simply provided with a local settlement cue. This situation may arise due to active inhibition of the sensory system by which the larva responds to local settlement cues (summarized in [87]), but this type of inhibition can be overcome by an appropriate environmental trigger (in the case of precompetent echinoid larvae, a brief exposure to turbulence) that modifies the reactivity of larvae to more localized chemical or tactile cues.
— *Phase 3: competent larva*. At this stage, the larva can respond to local settlement cues and settle. During the competent period, if it is a so-called ‘desperate’ larva [64,65], then the nature of its response to settlement cues can change (i.e. it can get less choosy) during the competent phase, culminating in larvae that will settle spontaneously (in the complete absence of a cue). If it falls into the ‘death before dishonour’ class [61], then its response to settlement cues does not change during the competent period.

In optimally developing *D. excentricus* larvae at approximately 21°C, Phase 1 begins at hatching on day 1 and lasts until about day 7 or 8; Phase 2 begins on about day 8 or 9 and lasts until about day 11 and Phase 3 (in the absence of previous turbulence exposure) begins on about day 11 or 12. A broader evaluation of this three-phase conception of larval development awaits further tests with combinations of inducers throughout development in a wide range of marine invertebrate larvae, as well as in comparable stages in non-invertebrates.

### 4.5 Comparative approaches

Taken as a whole, the findings we report here alongside our previously published data on purple sea urchins [16] paint a tantalizing portrait of evolution shaping how larvae respond to environmental information (in this case turbulence) so as to maximize their likelihood of settling at the appropriate place and time. Our comparative data to date are limited to two species—*S. purpuratus* and *D. excentricus—*with an evolutionary divergence time of more than 250 Mya [88]. The evidence for similar turbulence responses in both of these echinoderms could indicate a conserved effect, especially if analogous responses can be quantified in a broad range of echinoid and other echinoderm taxa, but it is also possible that species response sensitivities could be tuned to turbulence intensities characteristic of their preferred adult habitat.

A targeted examination of the potential for evolutionary tuning of the turbulence response should involve an explicitly comparative approach [89], with independent contrasts of relatively closely related echinoid species pairs that differ in adult habitat characters. This type of comparative study would enrich our understanding of the impact that hydrodynamic forces have on shaping larval life histories. Coupled with our new and expanding conception of what it means for a larva to be competent, such an approach also would highlight marine larvae and their metamorphoses as a fruitful paradigm for synthesizing ecology, physiology and developmental approaches in our efforts to understand the evolution and diversity of life in the sea.

## 5. Conclusion

Competence in marine animals has been operationally defined as the point during ontogeny when larvae first become responsive to localized settlement cues and transform to the juvenile stage. Despite its apparent commonality, a mechanistic understanding of what it means for larvae to be competent has remained elusive. Here we demonstrate that the transition from precompetence to competence is not as discrete as has often been assumed. A brief exposure to turbulence of an intensity comparable to what larvae would experience when transiting from offshore waters to the near shore will induce otherwise precompetent sand dollar (*D. excentricus*) larvae to immediately become operationally competent and respond to localized settlement inducers. This magnitude of turbulence exposure can shorten larval development by approximately 20%, which if true of shoreline species in general, would herald a substantial rethinking of our understanding of larval dispersal dynamics in the marine environment. Furthermore, we identify ontogenetic changes in responsiveness of larvae to turbulence as they proceed through the precompetent period. This observation indicates that the turbulence response can be moulded by natural selection, and may thus be an unappreciated contributor to patterns of species distributions, as well as to the persistence and recovery of marine populations over time. Based upon our results, we offer a modified conception of precompetence and competence, and suggest that this new concept could be tested in a variety of animal and non-animal dispersive stages in the ocean.
